# Analyze the Effect of Steaming on the Chemical Constituents, Defecation and Liver Injury of Polygonum Multiflorum Radix (Heshouwu) by Multiple Analysis Techniques Combined with Multivariate Statistics

**DOI:** 10.3390/molecules27196284

**Published:** 2022-09-23

**Authors:** Xiaolei Du, Lili Xu, Zhe Zhang, Yang Wang, Huifen Li, Weiliang Cui, Huibin Lin

**Affiliations:** 1 School of Pharmacy, Shandong University of Traditional Chinese Medicine, Jinan 250355, China; 2 Shandong Institute for Food and Drug Control, Jinan 250101, China; 3 Shandong Academy of Chinese Medicine, Jinan 250014, China

**Keywords:** Polygonum multiflorum radix, Polygoni multiflori radix praeparata, steaming, chemical constituents, defecation, liver injury

## Abstract

Steaming is a characteristic pharmaceutical skill in Traditional Chinese Medicine (TCM). Polygonum multiflorum radix (PM) and its steamed products have been used in Asia for centuries. Raw Polygonum multiflorum radix (RPM) is commonly used to promote defecation but can exert toxicity, especially in liver injury. However, RPM can be made converted into Polygoni multiflori radix praeparata (PMP) by steaming; this is considered a good method to reduce defecation and liver injury caused by PM in Asia. The chemical constituents of TCM are the key to its action. We systematically analyzed the effect of steaming on PM constituents, defecation, and liver injury. We identified 13 main constituents from PM and PMP; the results showed that after being steamed, two constituents (TSG, catechin) had decreased, six constituents (such as procyanidin B1 or B2) had disappeared, four constituents (such as emodin, physcion) had increased, emodin-8-O-β-D-glucoside remained unchanged in PMP. Pharmacological experiments showed that PM could promote defecation; however, there were no obvious effects in response to PMP. Only a high dose of PM for 14 days caused some degree of liver injury, although this injury disappeared after 14 days of drug withdrawal. Network pharmacology and molecular docking studies showed that TSG, emodin and physcion were the most effective in promoting defecation and causing liver injury. Collectively, our findings show that steaming can reduce the effect of PM on promoting defecation and reducing liver injury. TSG may be one of the important constituents in PM that can promote defecation and cause liver injury.

## 1. Introduction

Polygonum multiflorum radix (PM, Heshouwu in China), originated from the root of *Polygonum multiflorum* Thunb, has been used in Asia for thousands of years as a common tonic. However, in recent years, the number of reports relating to liver injury caused by PM has increased, thus attracting worldwide attention. Some studies have suggested that emodin, physcion, and other five major free anthraquinones (such as aloe-emodin, rhein, and chrysophanic acid) in the aqueous extract of PM are the leading causes of liver injury [1,2,3,4,5,6,7]. Furthermore, the repeated administration of emodin in large doses can accumulate in liver cells, thus causing liver injury [8]. Anthraquinones and stilbene glycosides are the main metabolites of PM; long-term use of PM will lead to the accumulation of emodin, TSG, aloe emodin-8-glucoside and other constituents in the kidney, liver and bile, resulting in liver injury [9,10]. It has been reported that emodin-8-O-β-D-glucoside plays a key role in the idiosyncratic liver injury caused by PM and acted synergistically with TSG to cause more severe liver injury [11]. However, some scholars believed that tannins in PM caused liver injury in rats when used at high doses over long periods, some liver injury at medium doses over the short term, and no significant liver injury when used in small amounts [12]. Thus, anthraquinones, stilbenes glycosides [13] and tannins [14] are considered to be toxic constituents of PM; however, these views are not universally accepted.

Processing is a characteristic pharmaceutical skill in China which significantly discriminates western medicine from TCM. Processing includes frying, steaming with water or rice wine, and braising with rice wine or liquorice liquids [15]. The processing of TCM is a process that causes quantitative and qualitative changes in the constituents, such as changes in drug properties, primary functions and the adjustment of clinical application [16].

In China, natural plants, animals and minerals must be strictly processed before use in TCM [15]. Appropriate processing may reduce toxicity, drastic properties, and side effects, promote therapeutic effects and modify the nature and action [17].

TCM science states that raw drugs and processed products act differently. PM is one such classic drug. PM is commonly applied to defecation, whereas Polygoni multiflori radix praeparata (PMP, Zhiheshouwu in China) is used more frequently in practice mainly because of its tonicity, its ability to promote hair growth and its anti-aging effects [18]. Modern studies have proved that processing can reduce PM’s laxative effects and ability to cause liver injury [19]. Studies have also found that processing may change the constituents of PM [20,21,22,23]. In TCM theory, “toxic herbs” is the general term used for medicine. The Chinese population believes that the “toxicity” of TCM is the basis for curing diseases [24,25]. For example, as described by Lei Jing, “Drugs cure diseases because they are toxic, which means that drugs have the bias of the four Qi and five flavors of TCM. Drugs can avoid evil spirits and stabilize righteousness, so it is said that drugs with the bias of four Qi and five flavors TCM can strengthen the body’s resistance to eliminate pathogenic factors.” [26]. Thus, it is essential to study the relationship between changes in the chemical constituents and the pharmacological and toxicological effects of PM before and after processing to understand the relationship between the toxicity and efficacy of TCM.

In this study, we used ultra-high performance liquid chromatography with E-electrostatic sector (UHPLC-QE/MS) technology, a pharmacological strategy, network pharmacology and molecular docking technology to comprehensively analyze the similarities and differences between PM and PMP from three aspects (differences in the chemical constituents, laxative effects and liver injury) to provide the scientific basis for understanding the relationship between toxicity and efficacy of PM and PMP and to guide clinical precision medicine.

## 2. Results

### 2.1. Qualitative Analysis of Constituents

**Figure 1:**
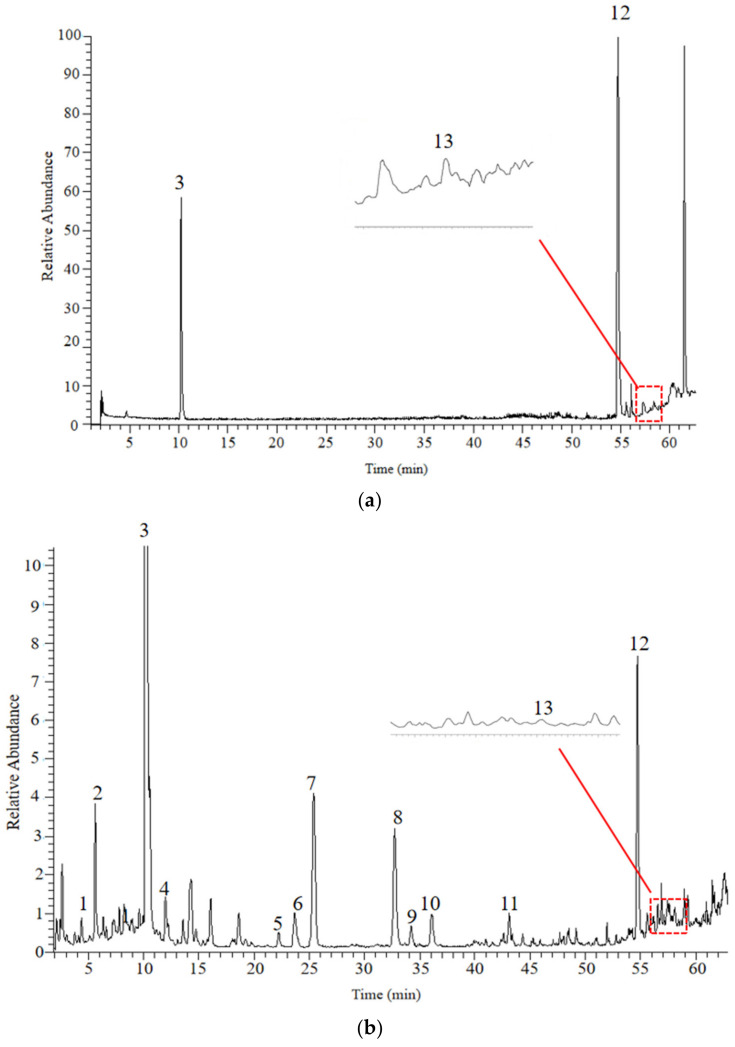

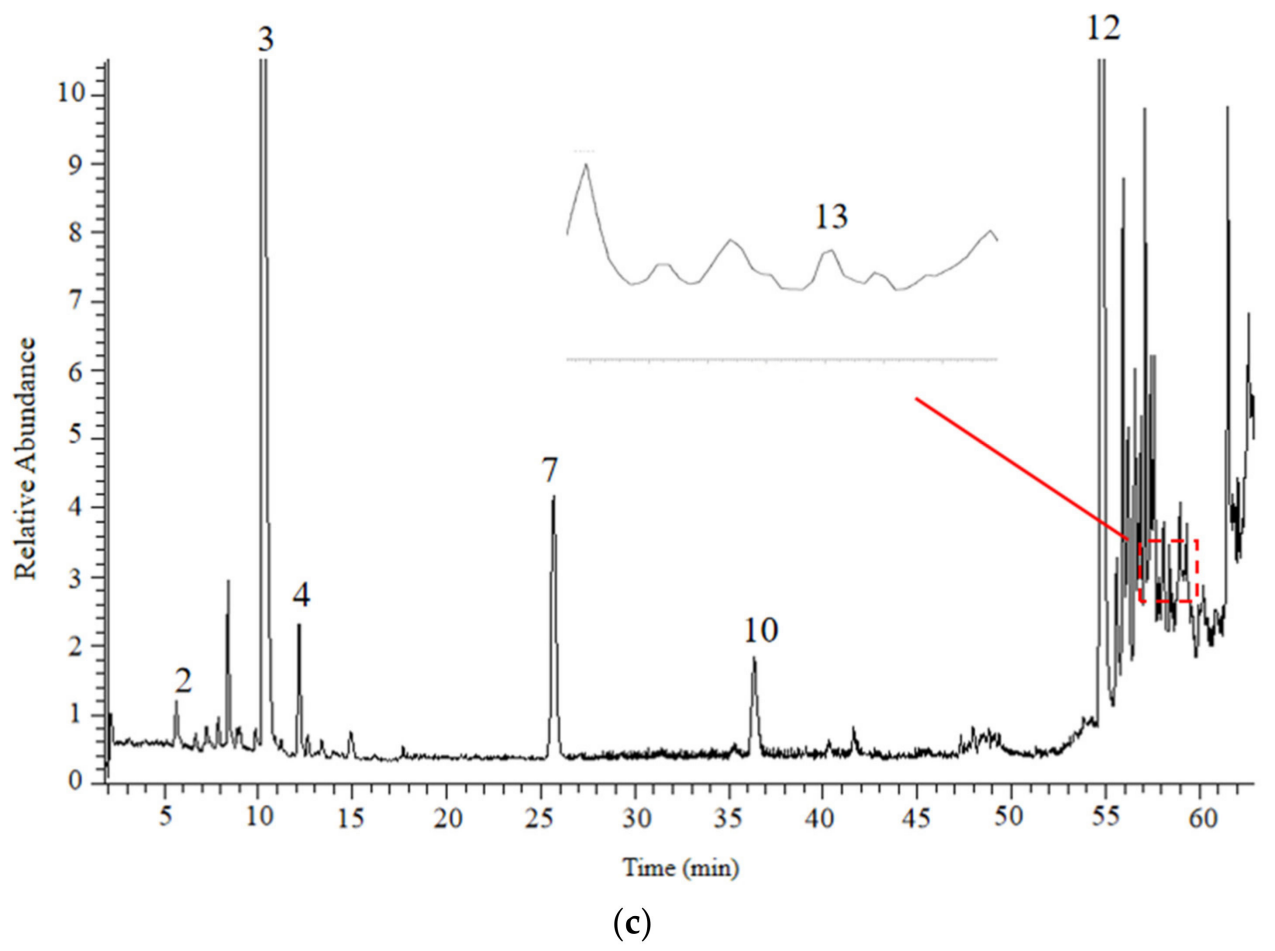
The BPCs of PM and PMP in the negative ion mode: mixed reference substance (**a**); PM in negative ion mode (**b**); PMP in negative ion mode (**c**).

We analyzed samples of PM and PMP with a UHPLC-QE/MS in both positive and negative ion modes. The representative basic peak chromatograms (BPCs) corresponding to negative signals generated by PM and PMP are shown in Figure 1. Details of the identified constituents are listed and presented in Table 1. Analysis showed that 13 constituents in PM exhibited different degrees of changes in PMP.

### 2.2. Observation of General Signs in Rats

During the period of administration, the rats in the control group were in good general health, there were no abnormalities of eating, drinking or defecation. The fur was white, smooth and shiny and there were no instances of infection or death. Compared with the control group, the rats in the Polygonum multiflorum high dose group (PM-H) began to show poor spirit, less activity and dull hair color on the 7th day of administration. As the administration time increased, these phenomena became aggravated and occasionally unformed feces were produced. Rats in the Polygonum multiflorum medium dose group (PM-M) showed effects that were similar to those in the PM-H group, although the onset of these effects occurred later; furthermore, these effects were milder. On the 12th day of administration, there was slightly poor spirit, slightly reduced activity, dark fur and no signs of abnormal defecation. Rats in the Polygoni multiflori Radix praeparata high dose group (PMP-H) consumed more food and drank more water, their fur was normal, mental state was normal and the levels of activity were higher than in the other groups. After seven days of administration, there was an increase in the number of feces particles; furthermore, the feces were slightly dry. Rats in the Polygoni multiflori Radix praeparata medium dose group (PMP-M) showed effects that were similar to those in the PMP-H group although these effects appeared later and were milder.

### 2.3. Effects of PM and PMP on Body Weight in Rats

**Figure 2:**
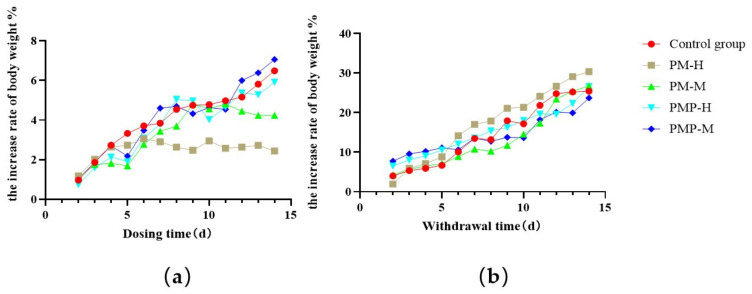
The increase in body weight in rats: during administration (**a**); during drug withdrawal (**b**).

Results are shown in Figure 2. In the control group, there was a steady increase in body weight. From day 2 to day 6 of PM-H, the increase rate of body weight increased slowly. From day 7 to day 14 of PM-H, the rate of increase in body weight did not change significantly. Rats in the PM-M group showed an increase in weight from day 2 to day 11 although this decreased slightly from day 12 to day 14. The body weight of rats in the PMP-H group also increased but was marginally lower than that of rats in the control group from day 12 to day 14 of administration. The increase in body weight in rats from the PMP-H group also increased and was slightly higher than that of the control rats from day 12 to day 14 of administration (Figure 2a).

During drug withdrawal, the rate of weight increase in each group showed an upward trend. In comparison, the rate of weight increase in rats from the control group decreased slightly on days 7 and 9 and continued to increase for the remaining period of time. On day 4, the weight gain of rats in the PM-H group was higher than that of the other groups and continued to increase. The weight gain of rats in the PM-M group was slightly lower than that of rats in other groups from day 5 to day 10 and decreased on day 7; subsequently, the rate of growth continued to increase. The rate of weight increase in rats in the PMP-H group continued to rise while that of rats in the PMP-M group decreased slightly from day 5 to day 10 and then continued to increase (Figure 2b).

### 2.4. Effects of PM and PMP on Liver Weight Index

**Figure 3:**
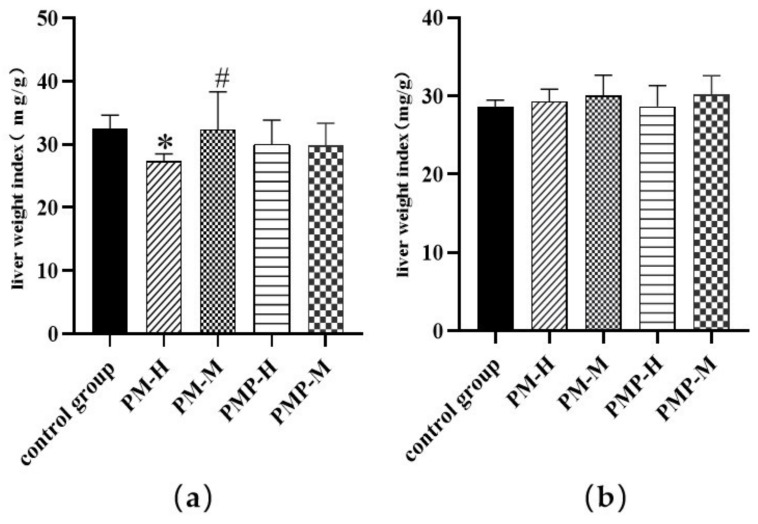
Liver weight index after drug administration (**a**); liver weight index after drug withdrawal (**b**); * significantly different from the control group, (*p* < 0.05); # significantly different from the PM-H, (*p* < 0.05).

As shown in Figure 3, compared with the control group, the liver weight index of rats in the PM-H group decreased significantly (*p* < 0.05); there were no significant differences between the other groups and the control group. Compared with PM-H rats, the liver weight index of PM-M was increased significantly (*p* < 0.05); this suggested that the administration of PM-H reduces liver weight. There was no significant difference in liver weight index after drug withdrawal, thus suggesting that the liver injury caused by the drug was repaired after stopping administration.

### 2.5. Effects of PM and PMP the Chromaticity of Feces

Taking the control group as an example, the same sample was measured repeatedly six times. The RSD (relative standard deviation) of L* (brightness), a* (red-green) and b* (yellow-blue) were 0.85%, 2.15% and 1.73%, respectively, indicating that the precision of the instrument was good. Then, we took six samples for measurement, the RSD of L*, a*and b* were 2.34%, 2.14% and 0.88%, respectively, thus indicating that the method has good repeatability.

**Figure 4:**
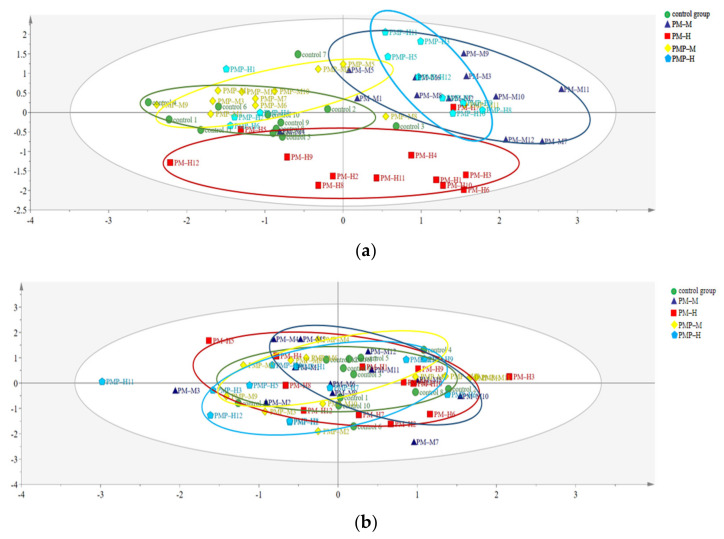
PCA model based on data of fecal chromaticity value: after administration (**a**); after drug withdrawal (**b**).

Following administration, the L*, a* and b* values of feces differed between groups; the drug had a significant influenced on the chroma value of feces (Table 2). A principal components analysis (PCA) model, an unsupervised pattern recognition technique, was established with R^2^ (X) (cum) = 1.00 and Q2 (cum) = 0.986. As shown in Figure 4a, the different dosing groups were mainly distributed in three regions.

The distribution area reflects the difference in the feces chromaticity. The overlapping distribution areas indicated no difference in feces, and the drug did not significantly affect feces. If the distribution area is scattered, there is a difference in the feces, and the drug substantially affects the feces. As shown in Figure 4a, the distribution areas of color difference values of the PMP-M and the control group overlapped, thus indicating that PMP-M treatment had little effect on the feces of rats. The PM-H, PM-M and PMP-H groups were far away from the distribution area of the color difference value for the feces of rats in the control group, thus indicating that the three groups had significant effects on the feces of rats. There was considerable overlap in the distribution area of color difference between the PM-M and the PMP-H groups, thus indicating that they had a similar effect on rat feces. The fecal chromaticity value of rats in the PM-H group was distributed in a single area; there was almost no overlapping area with other groups, thus indicating that the PM-H treatment had a significant effect on rat feces and was affected in different ways when compared to other drugs or doses.

After drug withdrawal and taking the control group as an example, the same sample was measured repeatedly six times. The RSD of L*, a* and b* were 2.81%, 2.35% and 2.03%, respectively, thus indicating that the precision of the instrument was good. Then we took six samples for measurement, the RSD of L*, a* and b*were 2.38%, 1.79% and 2.39%, respectively, thus indicating that the method has good repeatability.

Following drug withdrawal, there were no differences in the L*, a* and b* values for feces in each group, furthermore, the effect of drugs on the chromaticity of feces decreased (Table 3). A PCA model was established with R^2^ (X) (cum) = 1.00, Q2 (cum) = 0.956. As shown in Figure 4b, different dose groups were mainly distributed in the same area.

### 2.6. Effects of PM and PMP on Gastrointestinal Myoelectricity

**Figure 5:**
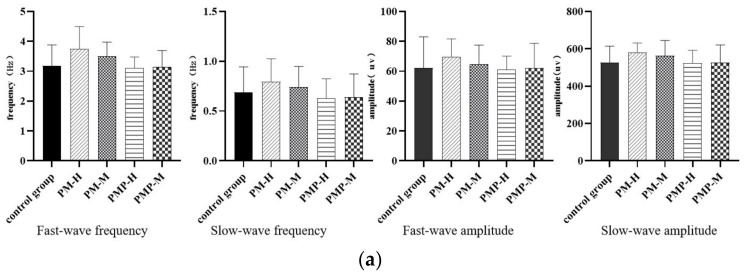

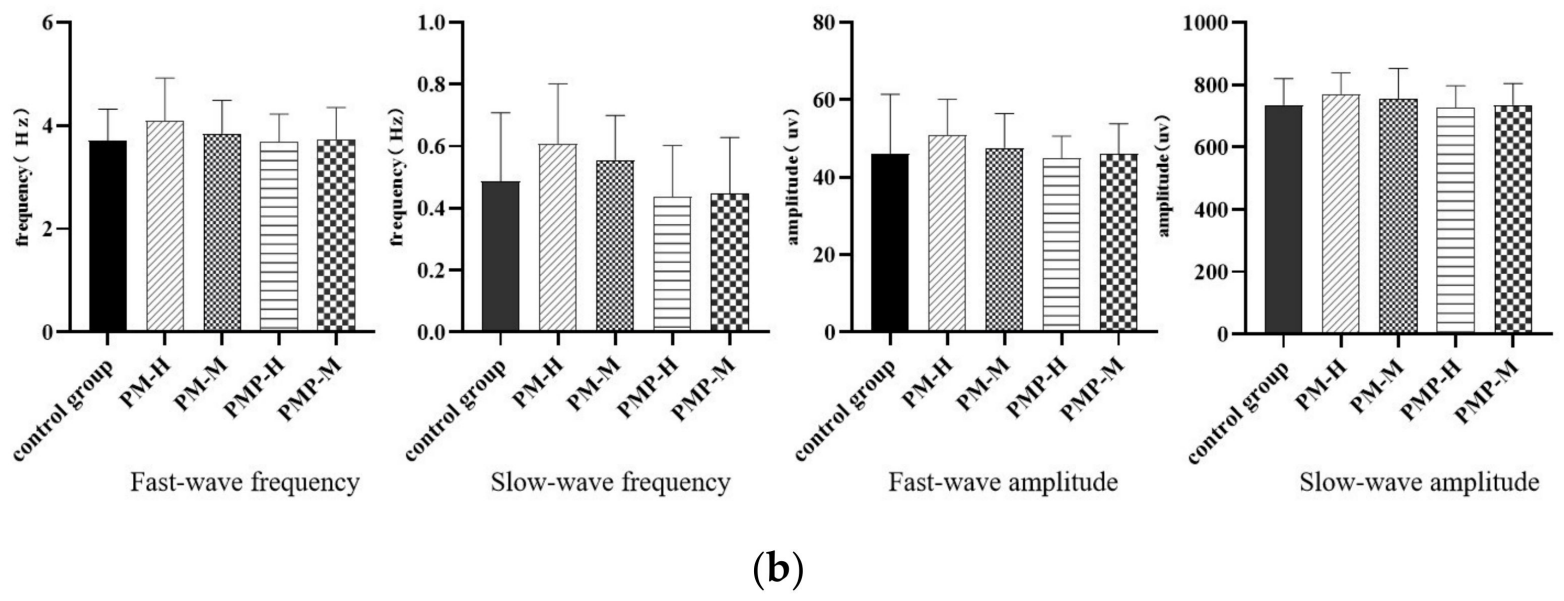
Gastrointestinal myoelectricity after administration: stomach (**a**) and duodenum (**b**).

Gastrointestinal myoelectric activity is the basis of gastrointestinal motility and can reflect the status of gastric emptying and intestinal transport. Compared with the control group, PM enhanced electrical fast-wave and slow-wave frequencies and amplitudes in the stomach and duodenum. PMP had no significant effect on gastrointestinal myoelectric signals (Figure 5), the gastrointestinal myoelectricity after administration are shown in Supplementary Materials, Figure S1. This suggested that PM can enhance the frequency and amplitude of gastrointestinal myoelectric signals, thus resulting in the disorder of gastrointestinal myoelectric signaling and promoting its effect on the gastrointestinal tract.

**Figure 6:**
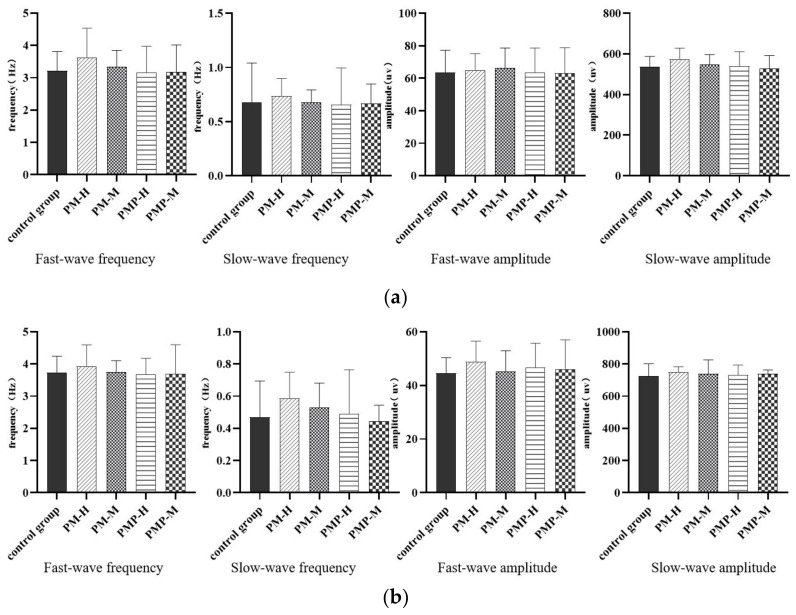
Gastrointestinal electromyography after drug withdrawal: stomach (**a**) and duodenum (**b**).

Compared with the control group, after drug withdrawal, PM still had an enhanced effect on gastrointestinal electrical frequency and amplitude, although this was not significantly different. PMP had no significant impact on gastrointestinal electromyography (Figure 6), The gastrointestinal myoelectricity after drug withdrawal are shown in Supplementary Materials, Figure S2. Compared with the amplitude and frequency of gastrointestinal electromyography during drug administration, the frequency and amplitude of gastrointestinal electromyography in the PM group decreased after drug withdrawal, thus suggesting that the effect of PM on the gastrointestinal tract can gradually recover to normality after drug withdrawal.

### 2.7. Effects of PM and PMP on Serum Liver Function Indexes in Rats

**Figure 7:**
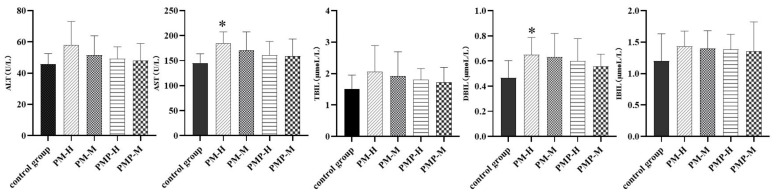
Serum liver function indexes after administration. * Significantly different from the control group, (*p* < 0.05).

**Figure 8:**
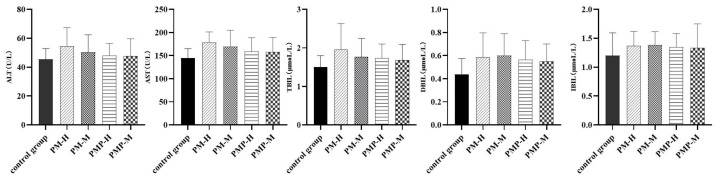
Serum liver function indexes after drug withdrawal.

Alanine aminotransferase (ALT), aspartate aminotransferase (AST), total bilirubin (TBIL), direct bilirubin (DBIL) and indirect bilirubin (IBIL) activity in serum or plasma are common clinical indicators of liver function, their levels of activity are a sensitive indicator of the degree of liver injury, with higher values indicating more severe liver injury. The serum level of each indicator was increased in each group when compared to the control group. Serum AST and DBIL were significantly increased in the PM-H group; this may have led to liver injury. The results are shown in Figure 7. Remarkably, after drug withdrawal the serum level in each group decreased, although this was still slightly higher than that in the control group. The results are shown in Figure 8.

These results indicated that PM and PMP had specific effects on the liver and bile excretion in experimental rats, and that large doses of PM had a significant impact. Following drug withdrawal, the liver function index gradually returned to normal.

### 2.8. Effects of PM and PMP on Liver Histopathology

The liver injury caused by PM, a common tonic herb, has received widespread attention over recent years. The drug-induced liver injury caused by PM is mainly hepatocellular; mixed types are rare. The histopathological analysis of liver sections after administration are shown in Supplementary Materials, Figure S3. The normal architecture of liver tissues in the control rats included hepatocytes, central veins, sinusoids and portal tracts (Figure S3a), the livers of rats in the PM-H group showed injury, including inflammatory cell infiltration, sinusoidal expansion and congestion. Rats in the PM-M, PMP-M and PMP-H groups did not show any initiation of liver injury. Following drug withdrawal, there was only slight hepatic sinus dilation in the PM-H group. No histopathological changes were observed in the other groups. The histopathological analysis of liver sections after drug withdrawal are shown in Supplementary Materials, Figure S4.

### 2.9. Screening of the Bioactive Constituents and Targets of PM

The main constituents of PM were imported into the Swiss Target Prediction database for target prediction. Then the UniProt database was used to convert protein targets into gene targets. After removing repetitive sequences, 17 active constituents were obtained, the results are shown in Table 4. Trans-feruloyltyramin, procyanidin B1, 3,3′-di-O-galloylprocyanidinb2 have anti-inflammatory, antioxidant, antibacterial, anti-melanogenesis, anticancer, anti-lipid and other biological activities [30]. In addition, 3,3′-di-O-galloylprocyanidinb2 protects against liver injury [31]. Catechin, gallic acid, and TSG have antioxidant, anti-ageing and liver protection effects [18]. Anthraquinones (emodin anthrone, citreorosein, chrysophanic acid, emodin-1-O-glucoside) have essential effects on antibacterial, anti-inflammatory, anti-ageing, improving memory and promoting gastrointestinal motility [9], chrysophanic acid also has antibacterial, anti-inflammatory, and diuretic properties [32]. Physcion, rhein, emodin and rhapontin have antibacterial, protecting the cardiovascular system and having a laxative effect [16,33,34]. Emodin-8-O-β-d-glucoside affects intelligence, is anti-ageing and promotes learning and memory [35]. Daucosterol are involved in immune regulation, cell differentiation, cell cycle and apoptosis and inhibits cancer cell proliferation [36]. The sterol components γ-sitosterol and β-sitosterol reduce cholesterol absorption, preventing cardiovascular diseases and anticancer [37].

A total of 133 potential targets for liver injury and 596 potential targets for constipation were obtained from the GeneCards database, OMIM database and Disgenet database.

### 2.10. Topology Analysis Results

**Figure 9:**
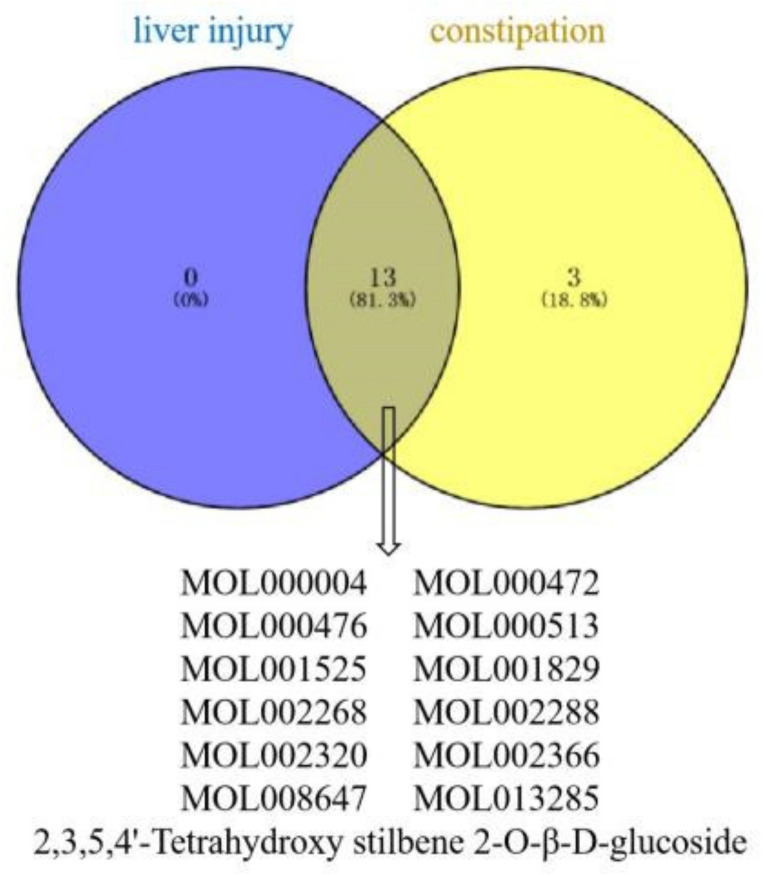
Venn diagram of constituents of PM for liver injury and constipation.

**Figure 10:**
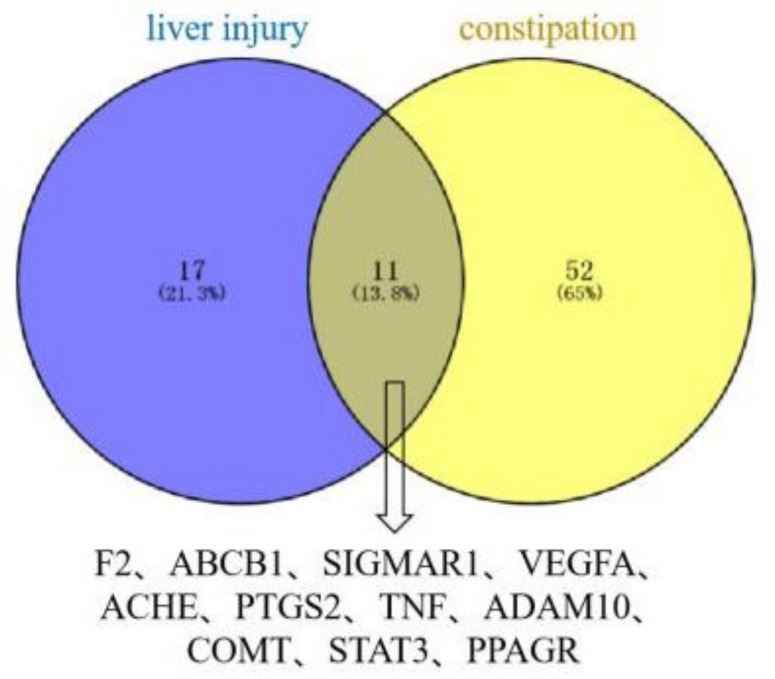
Liver injury-constipation intersectional target Venn diagram.

Of the 16 significant constituents of PM for the treatment of constipation, TSG, citreorosein, emodin, daucosterol, gallic acid, trans-feruloyltyramine, rhapontin, emodin-8-O-β-D-glucoside, γ-sitosterol, physcion, procyanidin B1, rhein and emodin-1-O-glucoside were the constituents that caused liver injury (Figure 9). A total of 11 potential targets for liver injury and the regulation of constipation by these constituents were identified (Figure 10).

### 2.11. Construction of a Mapping Protein-Protein Interaction (PPI) Network

**Figure 11:**
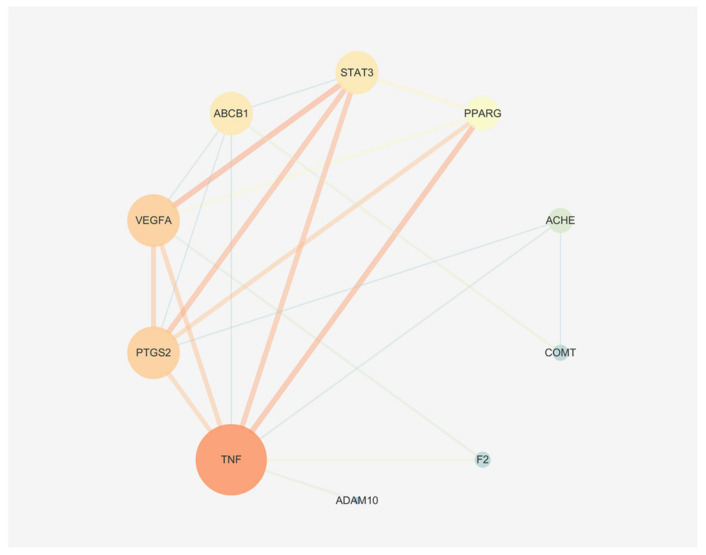
PPI network and node degree arrangement of common targets.

The PPI network diagram contained ten nodes and 24 edges (Figure 11), thus indicating a synergistic regulatory relationship between the intersectional targets of the screened intersectional constituents regulating liver injury and constipation rather than a single action regulation. CytoNCA topological analysis revealed target degree values in the following order: TNF > PTGS2 > VEGFA > STAT3 > F2 > ABCB1 > PPARG > ACHE > COMT > ADAM10.

### 2.12. Pathway Enrichment Based on Gene Ontology (GO) Analysis and Kyoto Encyclopedia of Genes and Genomes (KEGG) Databases

**Figure 12:**
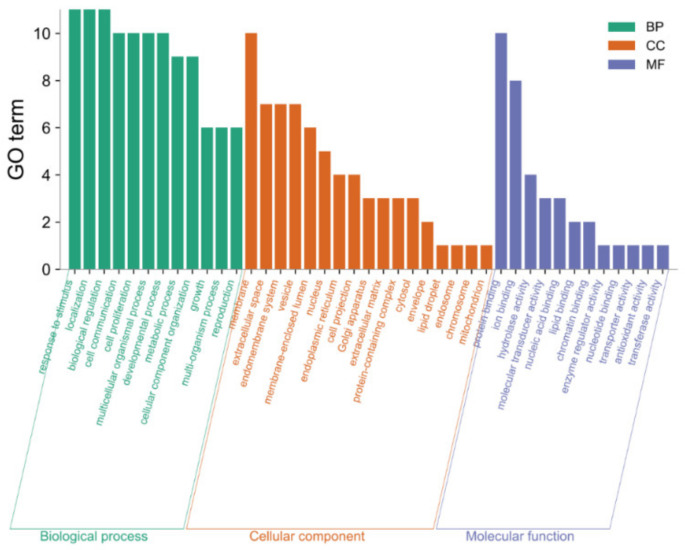
GO functional analysis diagram.

GO Webgestalt was used to analyze 11 core targets, and 40 results were obtained (Figure 12). Biological process (BP) analysis (shown in green) revealed the most significant enrichment in localization, response to stimulus and biological regulation. With regards to cellular constituents (CC) analysis (shown in orange), membrane was enriched in ten core targets. With regards to molecular functions (MF) analysis (shown in purple), protein binding was enriched in ten core targets, and ion binding was enriched in eight targets, thus indicating that the primary molecular functions of the targets were protein binding and ion binding.

**Figure 13:**
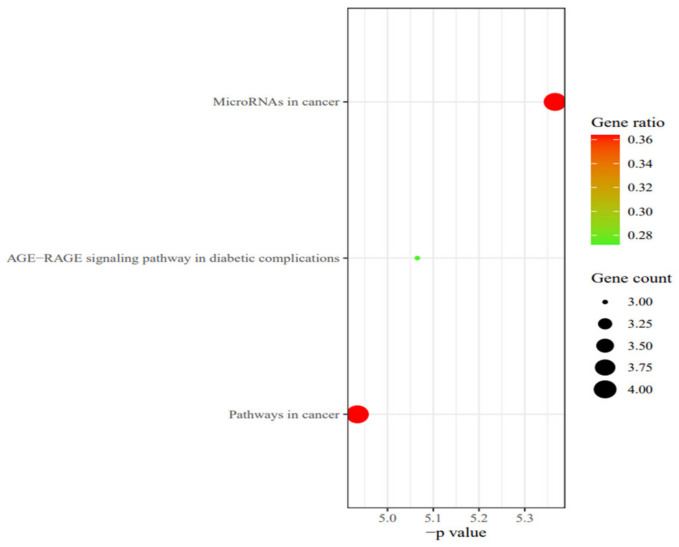
KEGG pathway enrichment analysis bubble map.

KEGG analysis of eleven core targets was performed by Meta scape; we identified three pathways according to *p* values: pathways in cancer, microRNAs in cancer, and AGE-RAGE signaling pathways in diabetic complications (Figure 13).

### 2.13. Molecular Docking

Network pharmacology results revealed the potential key targets of PM that cause liver injury and can be used to treat constipation. The key targets of PPI, TNF, PTGS2, VEGFA and STAT3 were molecularly docked with TSG, emodin, carotenol, gallic acid, trans-feruloyltyramine, emodin-1-O-glucoside, citreorosein, procyanidin B1, physcion, rhein, rhapontin, γ-sitosterol, emodin-8-O-β-D-glucoside to predict binding capacity and demonstrate the activity of the active constituents of PM against the relevant targets. It is generally believed that a binding energy less than −4.25 kcal/mol indicates that the ligand has a specific critical activity with the receptor, and that a binding energy less than −5.0 kcal/mol has excellent binding activity. A binding energy less than −7.0 kcal/mol has a robust critical activity. According to Table 5, except for procyanidin B1 and gallic acid, the binding energy was greater than −4.25 kcal/mol; the binding energy of the other eleven constituents was less than −4.25 kcal/mol. These results showed that all of the screened constituents exhibited specific binding activity with a target protein.

## 3. Materials and Methods

### 3.1. Chemicals and Materials

PM was obtained from Lunan Hope Pharmaceutical Group Corporation (Linyi, China); TSG (China National Institute for Food and Drug Control., Beijing, China), emodin (China National Institute for Food and Drug Control., Beijing, China), physcion (China National Institute for Food and Drug Control., Beijing, China); acetonitrile (Merck Co., Kenilworth, NJ, USA), ethanol (Tianjin Concord., Tianjin, China), formic acid (Nanjing Chemical Reagents Co., Nanjing, China).

ALT, AST, TBIL, DBIL and IBIL enzyme-linked immunosorbent assay kits (Jingmei Biotechnology Co., Ltd.; Jiangsu, China); 4% paraformaldehyde (Sangon Biotech Co., Ltd.; Shanghai, China).

### 3.2. UHPLC-QE/MS Analysis

PMP samples were prepared by steaming 4 times with water for 8 h each time and drying. PM and PMP samples were crushed into powder. An aliquot of 2 g homogenized sample powder was accurately weighed and heated to reflux for 1 h each time with 100 mL of ethanol/water (60:40, *v*/*v*) and 80 mL of ethanol/water (60:40, *v*/*v*) at room temperature, combine the two filtrates and volume to 250 mL. Then, the solution was filtered through a 0.22 μm filter membrane before analysis.

Detection was performed using a Vanquish FLEX ULTRA-high performance liquid chromatography with E-electrostatic sector (Thermo Fisher Scientific, Waltham, MA, USA). Chromatographic separation was performed with a water ACQUITY UPLC HSS column (2.1 mm × 100 mm, 1.8 μm; waters) maintained at 30 °C with linear gradient elution using (A) acetonitrile and (B) water (0.1% formic acid) as the mobile phase. The optimized gradient elution procedure was as follows: 10 to 22% A (0–7 min), 22 to 23% A (7–18 min), 23 to 27% A (18–33 min), 27 to 30% A (33–36 min), 30 to 36% A (36–42 min), 36 to 42% A (42–45 min), 42 to 50% A (45–51 min), 50 to 80% A (51–55 min), 80 to 90% A (55–68 min) and 90% A (58–63 min). The flow rate was 0.2 mL/min. The sample injection volume was 1 μL.

MS/MS identification was performed using a high-resolution mass spectrometer equipped with an electrospray ionization source using a quadrupole tandem electrostatic field track well. Ion mode: positive and negative ion mode; atomization voltage: 3.0 Kv; sheath gas flow rate: 35; auxiliary gas flow rate: 10; capillary temperature: 350 °C; auxiliary temperature: 350 °C; scan mode: full mass-DD MS2; scanning range: 200–1000. Sheath gas pressure; auxiliary gas pressure: 30, 40, 50 arb. Nitrogen was used as an atomizer and auxiliary gas. Data was obtained using Thermo Scientific Xcalibur.

### 3.3. Animals and Experimental Design

One hundred and twenty SD rats (50/50 male and female,190~220 g) were provided by Jinan Pengyue laboratory animal breeding co. Ltd. (Certificate No.: SCXK (Lu) 20190003, Jinan, China). All rats were raised on a regular rodent chow diet. The rats were kept at a constant temperature of 22 °C, relative humidity of 70%, and a light/dark cycle of 12 h:12 h dark/light. Rats were adapted for three days with free access to food and water. All of the procedures were in strict accordance with the China legislation on the use and care of laboratory animals and with the guidelines established by the Institutional Animal Ethics Committee and Committee for the Purpose of Control and Supervision of Experiments on Animals in China.

An aliquot of PM and PMP slices was accurately weighed and heated to reflux for 2 h each time with 10 times and 8 times of ethanol/water (60:40, *v*/*v*) at room temperature. The two filtered extracts were combined, alcohol was removed under reduced pressure and concentrated to a 108 mg/mL aqueous solution. The extract was stored at 4 °C before use for pharmacological experiments.

The experimental animals were randomly divided into five groups of 24 animals: control group, PM high dose group (PM-H), PM medium dose group (PM-M), PMP high dose group (PMP-H) and PMP medium dose group (PMP-M). According to the 2020 edition of the Chinese Pharmacopoeia, an adult dose of PM at 3 to 6 g, the animal equivalent dose was calculated using the following formula: rat dose (g/kg) = human dose (g/kg) × Km factor (the conversion factor). The gavage dose was 540 mg/kg/d for the middle dose group, and the gavage dose for the high dose group was three times higher than that of the middle dose group at 1620 mg/kg/d. To compare PM and PMP’s toxicological and pharmacological effects with the same dose on rats, the doses of the medium and high dose groups of PMP were consistent with those of the PM.

The rats in the medium-dose group were perfused with 36 mg/mL extract of PM and PMP once a day, which was equivalent to the human clinical dosage. The rats in the high-dose group were perfused with 108 mg/mL PM, and PMP extract once a day, which was equivalent to 3 times the human clinical dose; the rats in the control group were given 3 mL of normal saline once a day. After 14 days of the administration, fasted for 24 h, the related indexes of half of the rats in each group were determined. The remaining animals in each group were fed normally and fasted for 24 h on the 14th day after drug withdrawal. The relevant indexes were determined to observe the natural recovery of animals.

The rats were observed daily at regular intervals for signs of appearance, behavior and activity, changes in fur, shape and color of feces and the presence of abnormal secretions. The weight of the rats in each group was weighed at regular intervals from day 1 to day 28 of the experiment. The daily body weight growth rate was calculated: rats’ bodyweight growth rate of rats on day N of dosing = (body weight of rats on day N of dosing-body weight of rats on day 1 of dosing)/body weight on day one 1 of dosing × 100%. The high-quality COLORIMETER (Wave Optoelectronics Technology Co., Ltd.; Shenzhen, China) was used to measure the feces, get L*, a*, b* and the total color value E*ab which was obtained by the following formula: E*ab = (L^2^ + a^2^ + b^2^)^1/2^.

The rats in each group were anaesthetized by intraperitoneal injection of 10% chloral hydrate. The Biological Function Experiment System (BL-420N+, Taimeng Software Co., Ltd.; Chengdu, China) was used to measure the gastrointestinal myoelectric; blood was collected from the abdominal aorta, blood samples were left for 1 h, centrifuged at 5000 rpm for 10 min to obtain the supernatant by high-speed tabletop centrifuges (H3-18KR, Kecheng Instrument Equipment Co., Ltd.; Hunan, China), managed and stored at −80 °C for later use. Serum ALT, AST, TBIL, DBIL, IBIL levels were measured by ELISA kits (Jingmei Biotechnology Co., Ltd.; Jiangsu, China). Weigh the intact liver and calculate the relative weight index of the rat liver: relative weight index of the organ = total weight of the organ/body weight. The liver was rinsed with 0.01 mol/L PBS, 1 mm × l mm × l mm tissue was taken, fixed in 4% paraformaldehyde solution, paraffin sectioned, and HE stained to observe the structure of liver tissue under the light microscope for histopathological examination.

All data are reported as mean ± SD values. The one-way ANOVA and the post hoc least significant difference were carried out using SPSS 22.0 statistical software (SPSS, Inc., Chicago, IL, USA). A *p* value of less than 0.05 was regarded as being practical. PCA was performed using SIMCA 14.0 software (Umetrics, Inc., San Jose, CA, USA). The graphs were drawn using GraphPad Prism 8.0 software (GraphPad Prism, Inc., San Diego, CA, USA).

### 3.4. Pharmacology and Molecular Docking Studies of the PM Network

#### 3.4.1. Screen of Bioactive Constituents and Targets of PM

The TCM and Chemical Constituents database (http://www.organchem.csdb.cn/scdb/main/tcm_introduce.asp?nCount = 18757698) (accessed on 24 July 2022), BATMAN-TCM database (http://bionet.ncpsb.org.cn/batman-tcm/) (accessed on 24 July 2022) and TCMSP databases (http://tcmspw.com/index.php) (accessed on 24 July 2022) were used to collect the active ingredients of PM. The collected ingredients were screened with OB ≥ 30% and DL ≥ 0.18 and the inclusion of gallic acid, physcion, emodin, chrysophanic acid, citreorosein, rhapontin, emodin-1-O-glucoside and other active constituents that do not meet the requirements of OB and DL but have the basis in literature and experimental studies.

The Swiss Target Prediction database (http://www.swisstargetprediction.ch/) (accessed on 24 July 2022) was used to predict potential target active ingredients. “Liver injury” and “constipation” were used as keywords, and the Gene Cards database (https://www.genecards.org/) (accessed on 24 July 2022), Disgenet database (https://go.drugbank.com/) (accessed on 24 July 2022) and OMIM database (https://www.omim.org/) (accessed on 24 July 2022) were used to retrieve targets associated with liver injury and constipation. The UniProt database was used to standardize the targets, and the 2D and 3D structures of the targets were downloaded.

The potential targets for liver injury and constipation were obtained by repeated screening of disease targets and predicted targets of the potential active ingredients.

#### 3.4.2. Topology Analysis

The constituents involved in constipation and liver injury were visualized by Venny 2.1.0 online analysis tool (https://bioinfogp.cnb.csic.es/tools/venny/) (accessed on 24 July 2022), and then the target proteins of the selected constituents causing liver injury and constipation were visualized.

#### 3.4.3. PPI Networks

The String database (https://string-db.org/) (accessed on 24 July 2022) contains a large number of PPI relationships, and the potential targets were imported into the String database to obtain comprehensive PPI data. To further analyze the interaction relationship between targets and screen the key potential targets, the PPI data obtained were imported into Cytoscape 3.7.2 software to build a network, and the related topological parameters of the network were analyzed.

#### 3.4.4. GO Analysis and KEGG Pathway Analysis

GO analysis of protein functions into BP, CC, and MF were performed using the Web Gestalt online analysis tool (http://www.webgestalt.org/) (accessed on 24 July 2022). The pathway enrichment analysis results were mapped using MicrobioInfo (http://www.bioinformatics.com.cn/) (accessed on 24 July 2022).

KEGG pathway enrichment analysis was performed using the Meta scape database (https://metascape.org/gp/index.html#/main/step1) (accessed on 24 July 2022), “species” limited to “H. Sapiens”, and the system automatically enriched based on *p* value size. Bubble maps of pathway information were created using the Sanger Box database (http://sanger box.com/Index) (accessed on 24 July 2022) analysis platform.

#### 3.4.5. Molecular Docking Simulations

The 3D structures of the PM fractions were obtained from the PubChem database (https://pubchem.nc-bi.nlm.nih.gov/) (accessed on 24 July 2022) and Chem3D 19.0. The protein conformation was received from the PDB database (https://www.rcsb.org/) (accessed on 24 July 2022), and the water molecules and small molecule ligands were removed using Pymol software. Hydrogen bonding was added by Auto Dock Tools software for pre-processing, and the charge was calculated. Molecular docking of the receptor and ligand was performed, and the docking score value evaluated the binding activity.

## 4. Conclusions

In this study, the constituents in the PM and PMP were quickly separated and analyzed by liquid mass spectrometry. According to the reference substance location, mass spectrometry data, database matching technology and the relevant literature, we found that compared with PM, two constituents (TSG, catechin) had decreased, six constituents (procyanidin B1 or B2, tetrahydroxystyrene-2-O-(feruloyl)-hexose, aloe-emodin-8-O-glucoside, emodin-O-(malonyl)-hexglycoside, aloe emodin-8-O-(6′-O-acetyl)-β-D-glucoside, physcion-8-O-(6′-O-acetyl)-β-D-glucoside) had disappeared, four constituents (emodin, physcion, 2,3,5,4′-tetrahydroxystilbene2-O-β-D-(2″-O-monogallate)-glucoside isomer, physcion-8-O-β-D-glucoside isomer) had increased, and one constituent (emodin-8-O-β-D-glucoside) remained unchanged in PMP.

Animal experiments showed that PM could promote gastrointestinal motility and defecation while PMP had no significant effect on promoting gastrointestinal movement. PM-H will therefore have a certain impact on the liver of rats and cause liver injury. The other experimental groups showed no significant impact on the liver. The liver injury caused by PM-H can be repaired by the liver itself or after drug withdrawal. Compared with PM, the injury caused by PMP to the liver is not obvious, thus indicating that processing can indeed enhance the safety of PM. It has been reported that the liver injury caused by PM is related to the specific constitution of patients [38], There is no report relating to the effects of PMP on the liver injury in patients with a specific heterogeneous system. In this research, we aimed to investigate the effect of steaming on the toxicity and efficacy of PM. We did not use a specific heterogeneous animal model although this approach should be used in future research.

Using the screening of potential constituents of PM for liver injury and constipation treatment as an entry point, a total of 13 significant constituents were obtained that could cause liver injury and regulate constipation. Of these, TNF, PTGS2, VEGFA and STAT3 were critical proteins in the PPI network, these correspond to three significant signaling pathways. The 13 constituents can regulate constipation by regulating inflammatory response, immune response, angiogenesis and cell proliferation, and thus have some hepatotoxic safety risks. According to molecular docking technology, we predicted that TSG, emodin, physcion, citreorosein, rhein, γ-sitosterol, daucosterol, emodin-1-O-glucoside, trans-feruloyltyramine, emodin-8-O-β-D-glucoside, rhapontin, gallic acid and procyanidin B1 are the main constituents in PM with safety concerns related to target binding.

According to changes in the constituents and differences in the liver injury caused by PM before and after processing, and considering the results predicted by network pharmacology and molecular docking, steaming could indeed weaken the defecation effect of PM and also reduce liver injury. TSG, emodin and other constituents of PM are the most likely to cause liver injury.

## Figures and Tables

**Table 1:** Results of changes in the chemical constituents of PM after steaming.

Peak Number	Identification	Molecular Formula	Retention Time (min) in the Positive Ion Mode	[M + H]^+^ (*m/z*) (Error, ppm)	Fragment Ions in the Positive Ion Mode (*m/z*) b	Retention Time (min) in the Negative Ion Mode	[M–H]^−^ (*m/z*) (Error, ppm)	Fragment Ions in the Negative Ion Mode (*m/z*) b	Peak Area Change in PM
1	Procyanidin B1 or B2 [27]	C_30_H_26_O_12_	4.39	579.14874 (−2.609)	289.06918, 291.08667, 127.03886, 139.03868, 163.03857	4.41	577.13635 (2.297)	289.07199, 407.07809, 125.02336, 161.02351, 245.08192	Disappear
2	Catechin [27]	C_15_H_14_O_6_	5.63	291.08527 (−3.589)	123.04398, 139.03868, 147.04373, 205.08461, 207.06467	5.61	289.07198 (4.481)	109.02838, 125.02339, 151.03920, 203.07089, 245.08189, 289.07190	Decrease
3	TSG *	C_20_H_22_O_9_	10.17	407.13168 (−4.86)	199.07495, 245.08022, 227.06963, 151.03867, 107.04927	10.19	405.11905 (2.571)	243.06622, 225.05542, 201.05502, 272.33606, 137.02356	Decrease
4	2,3,5,4′-Tetrahydroxystilbene 2-O-β-D-(2″-O-monogallate)-glucoside isomer [28]	C_27_H_26_O_13_	11.96	559.14349 (−1.127)	153.01799, 171.02849, 245.07977, 315.07074	12.01	557.13104 (2.013)	125.02345, 169.01367, 243.06612, 313.05627, 395.64926	Increase
5	Tetrahydroxystyrene-2-O-(feruloyl)-hexose [27,29]	C_30_H_30_O_12_	ND	ND	ND	22.24	581.16754 (3.764)	175.03938, 193.05017, 225.05533, 243.06622, 337.09418, 405.12024	Disappear
6	Aloe emodin-8-O-glucoside [27]	C_20_H_24_O_9_	23.68	409.14835 (−2.343)	85.02892, 198.06767, 205.08568, 229.08548	23.67	407.13571 (5.038)	172.20393, 230.05818, 245.08182	Disappear
7	Emodin-8-O-β-D-glucoside [27]	C_21_H_20_O_10_	ND	ND	ND	25.39	431.09860 (3.078)	269.04568, 225.05540, 253.04970, 293.04559	Unchanged
8	Emodin-O-(malonyl)-hexglycoside [27]	C_24_H_22_O_13_	32.74	519.11273 (−2.187)	271.05957, 295.05981, 313.06992, 337.07007, 229.04907, 362.06958	32.73	517.09949 (2.465)	473.10901, 431.09958, 269.04581, 293.04523, 225.05540, 181.06358	Disappear
9	Aloe emodin-8-O-(6′-O-acetyl)-β-D-glucoside [27]	C_22_H_26_O_10_	ND	ND	ND	34.25	449.14633 (2.107)	215.03461, 230.05829, 245.08197	Disappear
10	Physcion-8-O-β- D-glucoside isomer [27]	C_22_H_22_O_10_	ND	ND	ND	36.07	445.11487 (1.947)	240.04274, 241.04623, 269.04379, 283.06146	Increase
11	Physcion-8-O-(6′-O-acetyl)-β-D-glucoside [27]	C_24_H_24_O_11_	ND	ND	ND	43.10	487.12537 (1.882)	240.04269, 283.06146, 292.03796	Disappear
12	Emodin *	C_15_H_10_O_5_	ND	ND	ND	54.70	269.04559 (1.14)	269.04578, 225.05547, 241.05052, 197.06030, 181.06537, 141.51163	Increase
13	Physcion *	C_16_H_12_O_5_	ND	ND	ND	58.39	283.06198 (6.642)	74.02315, 212.04640, 231.86180, 240.04262, 269.04538, 283.06131	Increase

“*” indicates comparison with reference substance.

**Table 2:** Fecal chroma value after administration (x ± s).

Group	L*	a*	b*	E*ab
control group	37.97 ± 2.72	8.12 ± 0.51	26.30 ± 2.39	47.01 ± 1.65
PMP-M	39.87 ± 2.30 ^##^^	8.03 ± 0.38 ^#^^^	26.10 ± 1.74 ^##^^ΔΔ^	48.39 ± 1.40 ^##^
PMP-H	41.96 ± 3.80 **^##^	7.88 ± 0.35 ^^^	23.06 ± 2.51 **	48.64 ± 2.90 ^##^
PM-M	42.41 ± 2.99 **^##^	7.46 ± 0.37 **	21.92 ± 0.37 **	48.37 ± 2.59 ^##^
PM-H	34.47 ± 2.70 **	7.60 ± 0.39 **	22.39 ± 2.46 **	41.89 ± 2.29 **

E*ab = (L^−2^ + a^−2^ + b^−2^)^−1/2^; * significantly different from the control group, (*p* < 0.05, *p* < 0.01); ^#^ significantly different from the PM-H, (*p* < 0.05, *p* < 0.01); ^^^ significantly different from the PM-M, (*p* < 0.05, *p* < 0.01); ^Δ^ significantly different from the PMP-H, (*p* < 0.05, *p* < 0.01).

**Table 3:** Fecal chroma value after drug withdrawal (x ± s).

Group	L*	a*	b*	E*ab
control group	37.87 ± 1.23	9.20 ± 0.56	29.40 ± 1.52	48.84 ± 1.27
PMP-M	37.81 ± 1.55	9.19 ± 0.63	29.83 ± 1.07	49.05 ± 1.32
PMP-H	37.15 ± 1.55	9.17 ± 0.54	30.64 ± 1.25	49.06 ± 1.05
PM-M	37.77 ± 1.41	9.16 ± 0.53	29.96 ± 1.76	49.10 ± 1.75
PM-H	37.66 ± 1.22	9.17 ± 0.53	28.91 ± 1.42	48.38 ± 1.41

E*ab = (L^−2^ + a^−2^ + b^−2^)^−1/2^.

**Table 4:** PM active constituents.

MOLID	Constituents	Oral Bioavailability (OB)	Drug Likeness (DL)
MOL008647	Trans-feruloyltyramine	86.71	0.26
MOL000004	Procyanidin B1	67.87	0.66
MOL000492	Catechin	54.83	0.24
MOL000096	3,3′-di-O-galloylprocyanidinB2	49.68	0.24
MOL002268	Rhein	47.07	0.28
MOL002288	Emodin-8-O-β-D-glucoside	44.81	0.8
MOL001525	Daucosterol	36.91	0.75
MOL002320	γ-sitosterol	36.91	0.75
MOL001987	β-sitosterol	33.94	0.70
MOL000513	Gallic acid	31.69	0.04
MOL003353	Emodin anthrone	24.72	0.21
MOL000472	Emodin	24.40	0.24
MOL000476	Physcion	22.29	0.27
MOL013285	Citreorosein	22.19	0.27
MOL001729	Chrysophanic acid	18.64	0.21
MOL002366	Rhapontin	18.31	0.82
MOL001829	Emodin-1-O-glucoside	10.35	0.80
--	2,3,5,4′-Tetrahydroxy stilbene 2-O-β-D-glucoside	--	--

**Table 5:** Binding activity of active constituents docked to target proteins.

Constituents	TNF (7jra)	TNF (5uui)	PTGS2 (5f19)	VEGFA (4qaf)	STAT3 (6njs)
TSG	−9.0	−4.9	−8.4	−5.3	−6.6
Emodin	−9.3	−5.0	−9.0	−9.2	−6.5
Physcion	−8.0	−4.8	−9.1	−9.1	−6.0
Citreorosein	−9.2	−5.0	−9.0	−8.8	−6.3
Rhein	−10.1	−5.0	−9.8	−9.2	−6.5
γ-sitosterol	−9.5	−4.9	−9.3	−9.3	−7.0
Daucosterol	−7.7	−4.5	−8.3	−7.7	−7.2
Emodin-1-O-glucoside	−6.7	−5.9	−9.8	−6.7	−6.7
Trans-ferulictyramide	−9.4	−4.6	−8.6	−8.2	−6.5
Emodin-8-O-β-D-glucoside	−7.1	−5.3	−10.0	−6.2	−6.7
Rhapontin	−6.8	−5.6	−8.9	−6.9	−6.7
Gallic acid	−6.4	−3.9	−6.5	−5.5	−4.9
Procyanidins B1	2.3	−5.2	−8.5	−6.6	−7.7

## Data Availability

Data is contained within the article or Supplementary Materials.

## Supplementary Materials

The following supporting information can be downloaded at: https://www.mdpi.com/article/10.3390/molecules27196284/s1, Figure S1: gastrointestinal myoelectricity after administration. Figure S2: gastrointestinal myoelectricity after drug withdrawal. Figure S3: histopathological analysis of liver sections after administration. Figure S4: histopathological analysis of liver sections after drug withdrawal.

## Sample Availability

Samples are not available from the authors.
