# Supplementary material for: Analyze the Effect of Steaming on the Chemical Constituents, Defecation and Liver Injury of Polygonum Multiflorum Radix (Heshouwu) by Multiple Analysis Techniques Combined with Multivariate Statistics
Source: Molecules. 2022 Sep 23;27(19):6284. doi: 10.3390/molecules27196284 (PMC9570837; doi:10.3390/molecules27196284)
Supplement: Supplementary file 1 [file molecules-27-06284-s001.zip › molecules-1905883-supplementary.pdf]

# Supplementary Materials

**Figure S1.** Gastrointestinal myoelectricity after administration: control group (**a**), PM-H (b), PM-M (**c**), PMP-H(d), PMP-M (e)

**Figure S2.** Gastrointestinal myoelectricity after drug withdrawal: control group (a), PM-H (b), PM-M (c), PMP-H(d), PMP-M (e)

**Figure S3.** Histopathological analysis of liver sections after administration (x 400): control group (a), PM-H (b),PM-M (c), PMP-H(d), PMP-M (e)

**Figure S4**. Histopathological analysis of liver sections after drug withdrawal (x 400): control group (a), PM-H (b), PM-M (c),PMP-H (d), PMP-M (e)

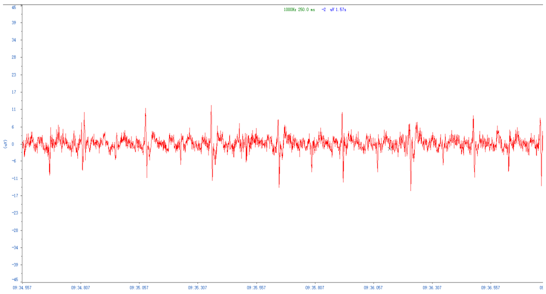

Fast-wave of stomach myoelectric

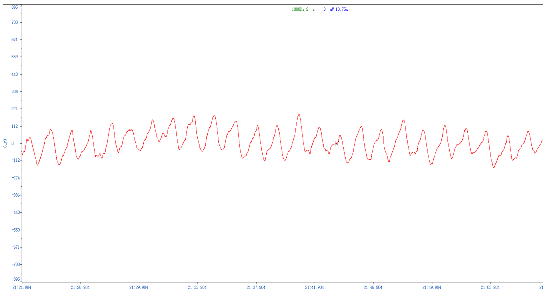

Slow-wave of stomach myoelectric

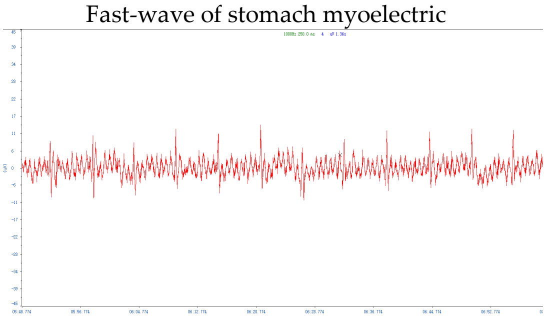

Fast-wave of duodenum myoelectric

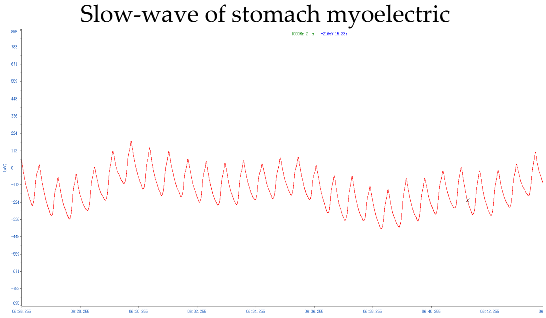

Slow-wave of duodenum myoelectric

(**a**)

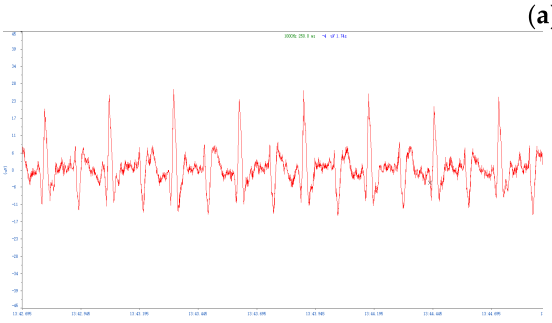

Fast-wave of stomach myoelectric

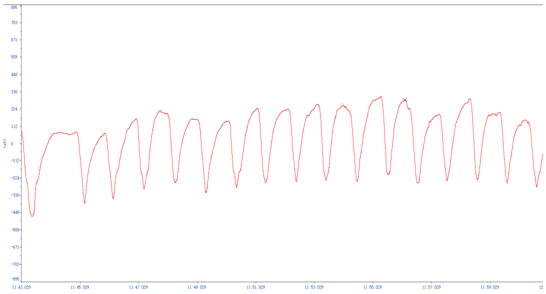

Slow-wave of stomach myoelectric

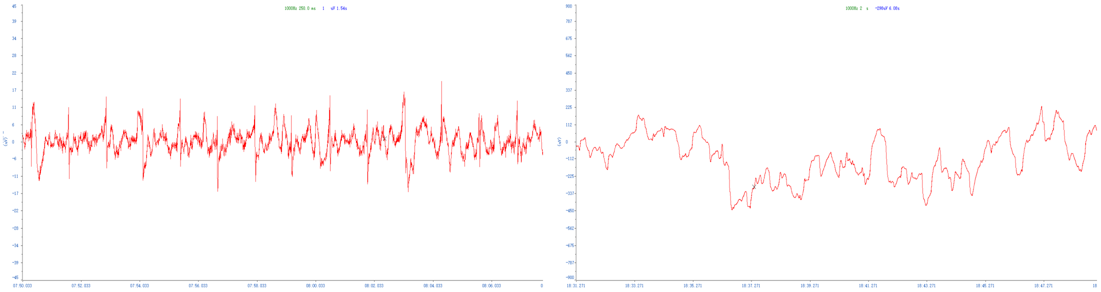

Fast-wave of duodenum myoelectric

Slow-wave of duodenum myoelectric

(**b**)

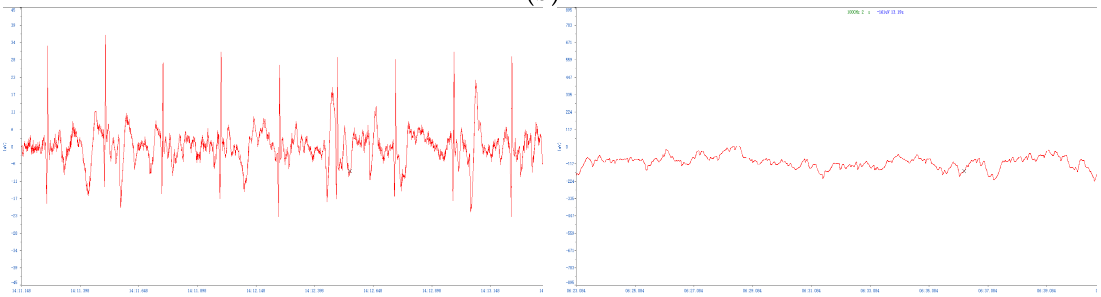

Fast-wave of stomach myoelectric

Slow-wave of stomach myoelectric

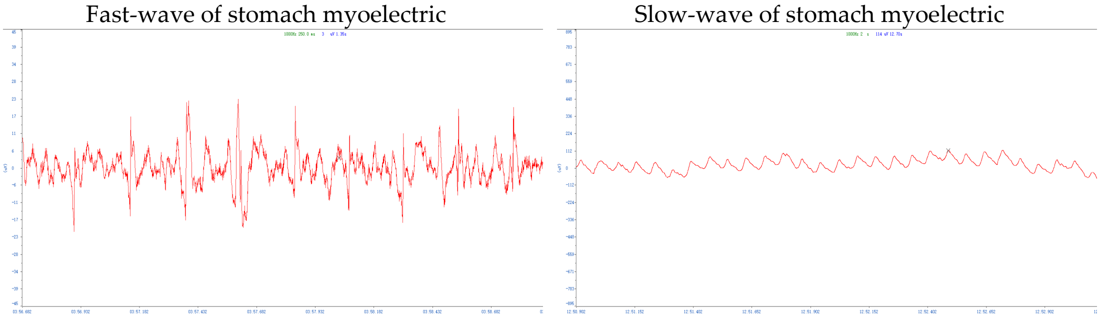

Fast-wave of duodenum myoelectric

Slow-wave of duodenum myoelectric

(**c**)

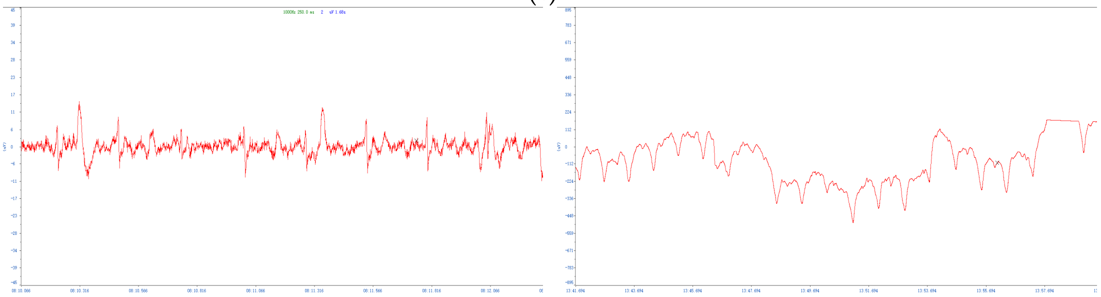

Fast-wave of stomach myoelectric

Slow-wave of stomach myoelectric

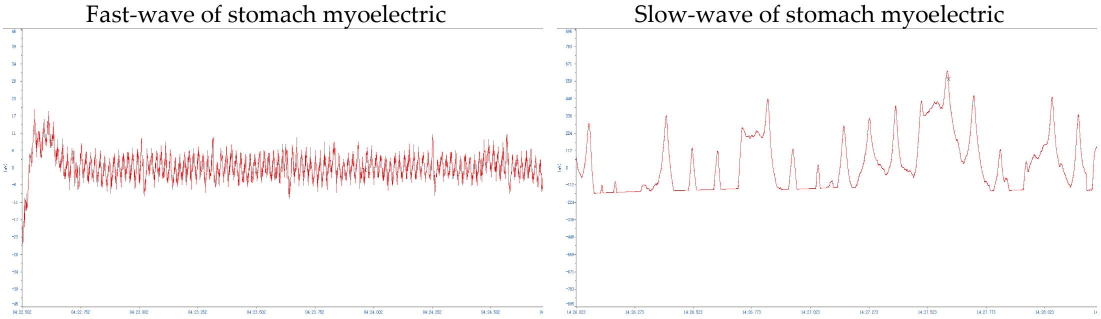

Fast-wave of duodenum myoelectric

Slow-wave of duodenum myoelectric

(**d**)

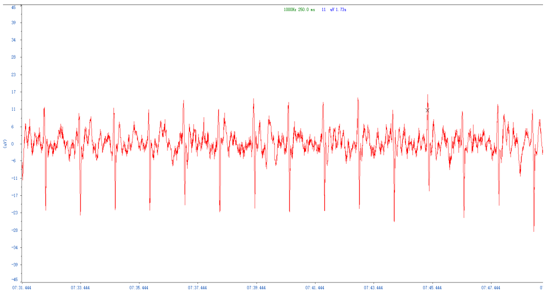

Fast-wave of stomach myoelectric

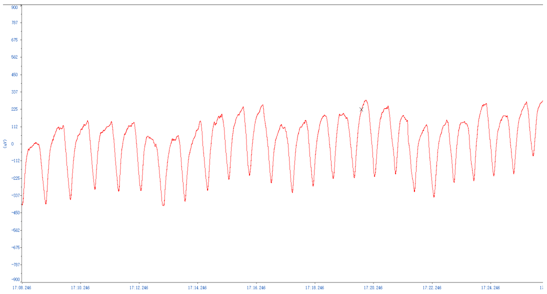

Slow-wave of stomach myoelectric

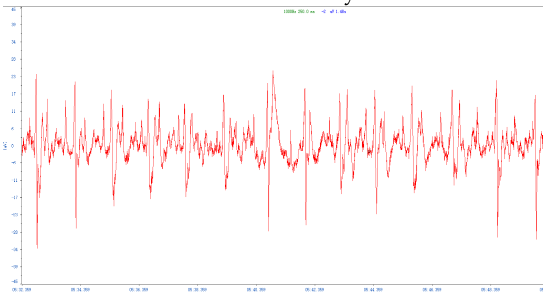

Fast-wave of duodenum myoelectric

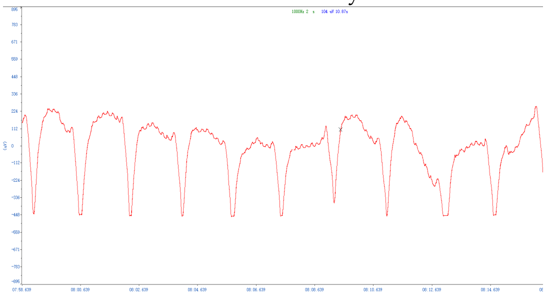

Slow-wave of duodenum myoelectric

(**e**)

**Figure S1.** Gastrointestinal myoelectricity after administration: control group (**a**), PM-H (**b**), PM-M (**c**), PMP-H(**d**), PMP-M (**e**)

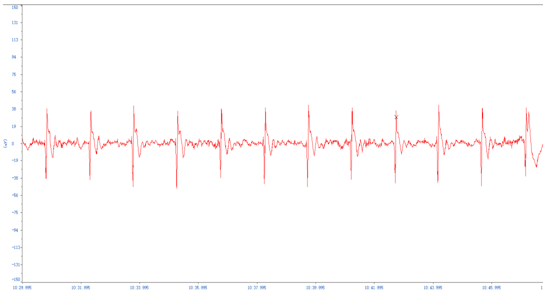

Fast-wave of stomach myoelectric

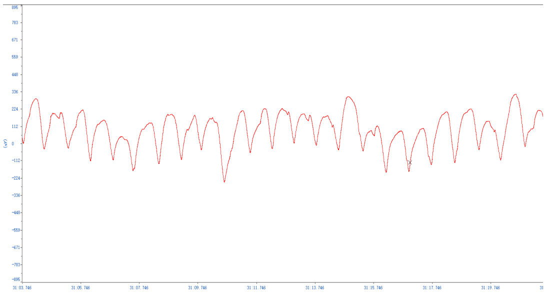

Slow-wave of stomach myoelectric

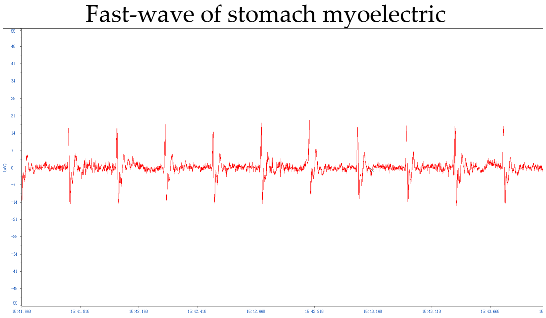

Fast-wave of duodenum myoelectric

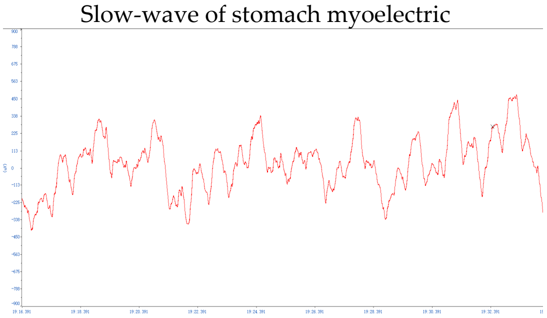

Slow-wave of duodenum myoelectric

(**a**)

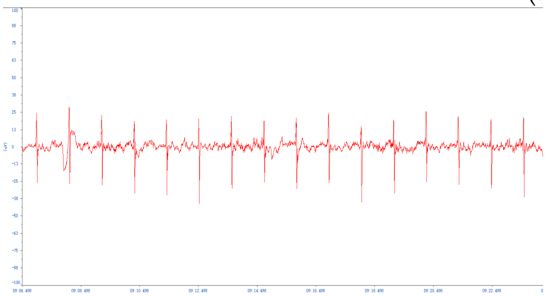

Fast-wave of stomach myoelectric

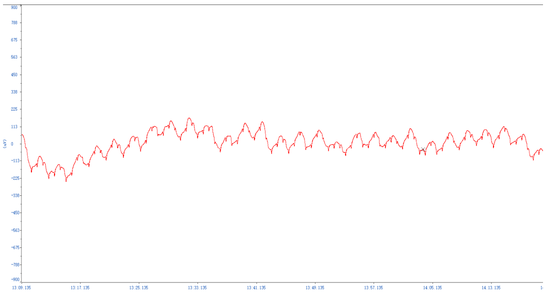

Slow-wave of stomach myoelectric

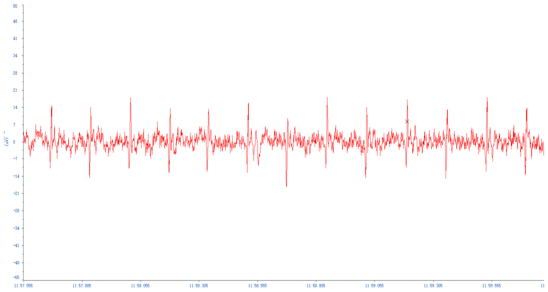

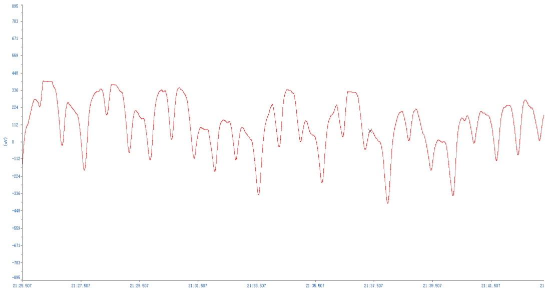

Fast-wave of duodenum myoelectric

Slow-wave of duodenum myoelectric

(**b**)

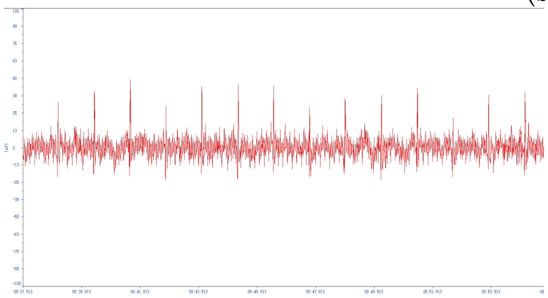

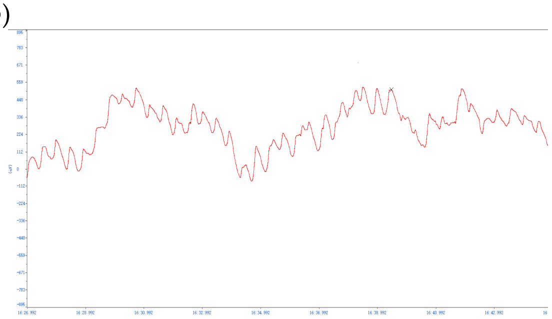

Fast-wave of stomach myoelectric

Slow-wave of stomach myoelectric

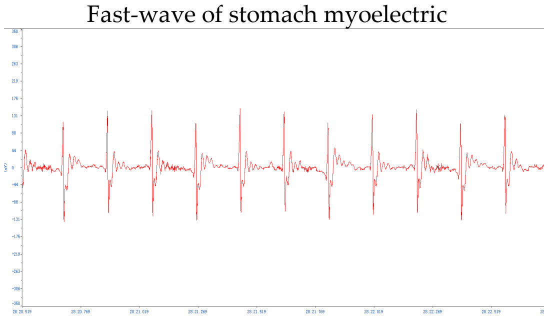

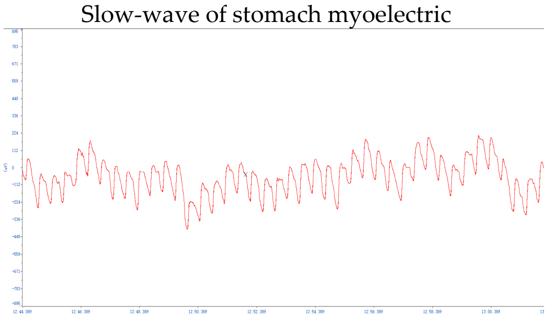

Fast-wave of duodenum myoelectric

Slow-wave of duodenum myoelectric

(**c**)

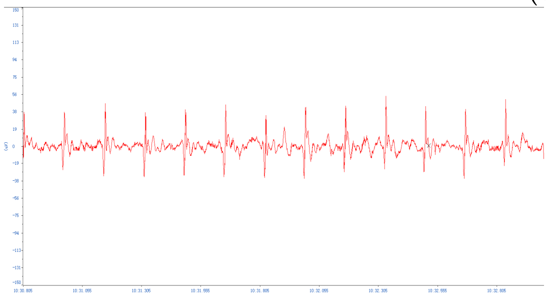

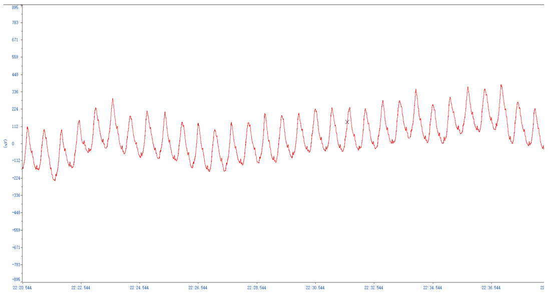

Fast-wave of stomach myoelectric

Slow-wave of stomach myoelectric

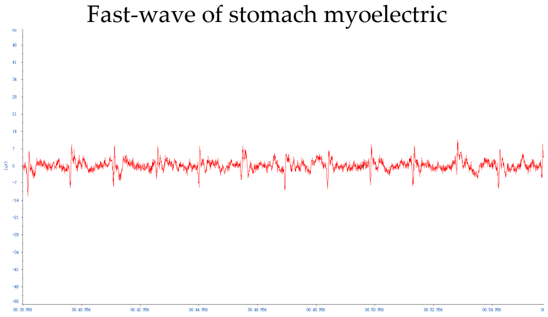

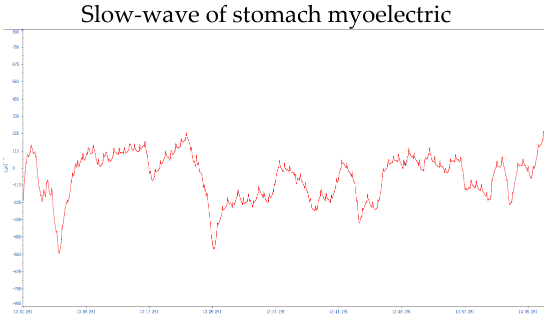

Fast-wave of duodenum myoelectric

Slow-wave of duodenum myoelectric

(**d**)

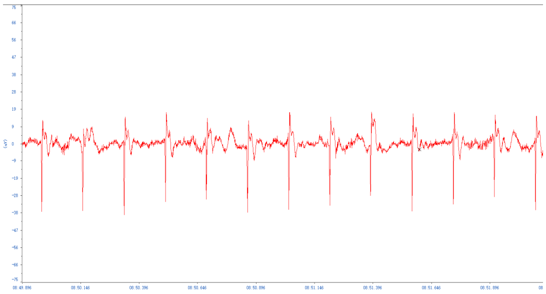

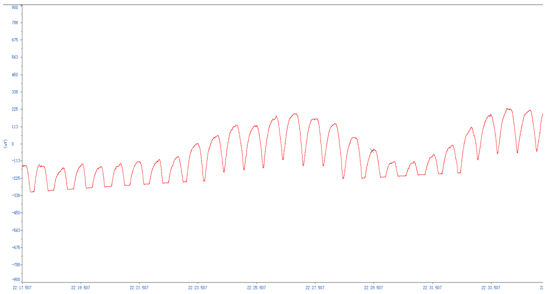

Fast-wave of stomach myoelectric

Slow-wave of stomach myoelectric

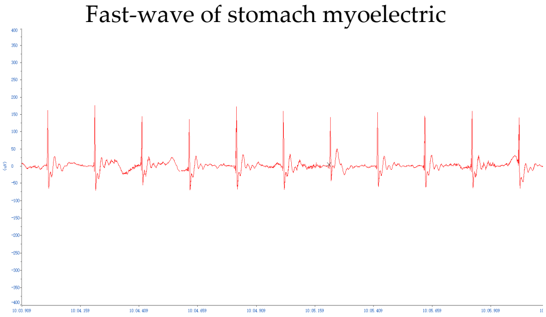

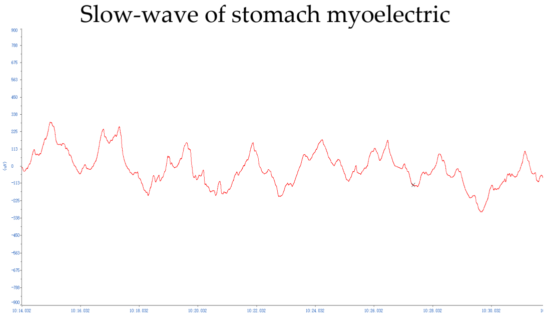

Fast-wave of duodenum myoelectric

Slow-wave of duodenum myoelectric

(**e**)

**Figure S2.** Gastrointestinal myoelectricity after drug withdrawal: control group (**a**), PM-H (**b**), PM-M (**c**), PMP-H(**d**), PMP-M (**e**)

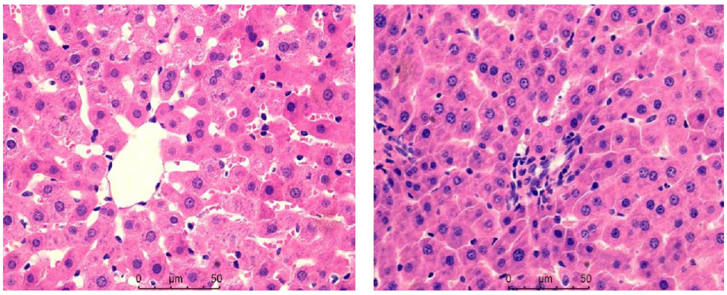


(**a**)

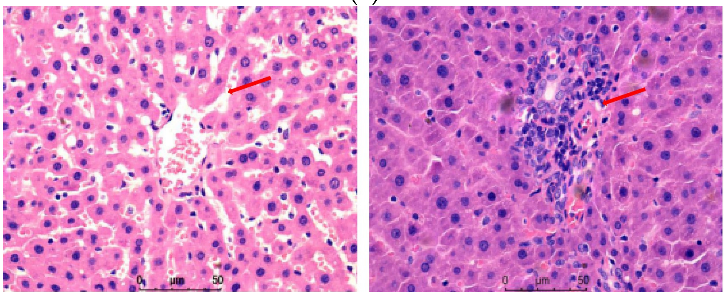


(**b**)

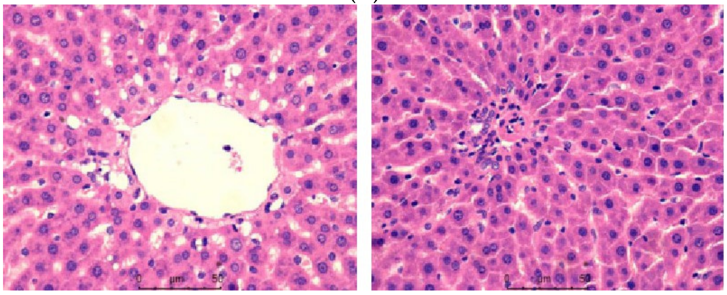


(**c**)

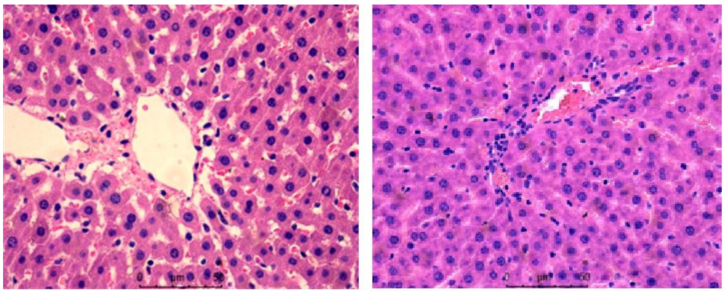

(**d**)

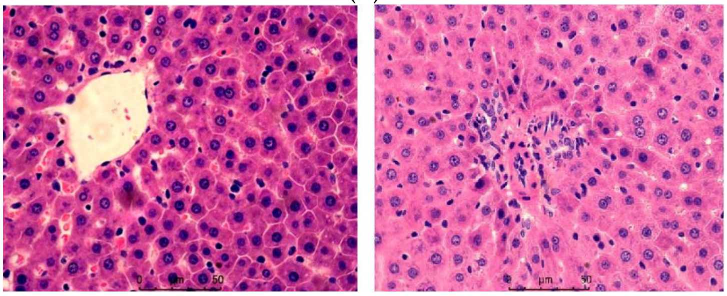

(**e**)

**Figure S3.** Histopathological analysis of liver sections after administration (x 400): control group (**a**), PM-H (**b**),PM-M (**c**), PMP-H(**d**), PMP-M (**e**)

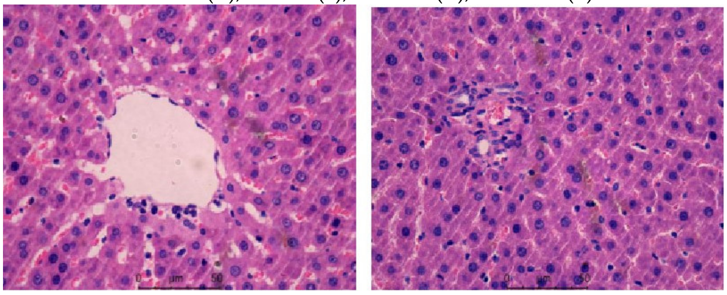

(**a**)

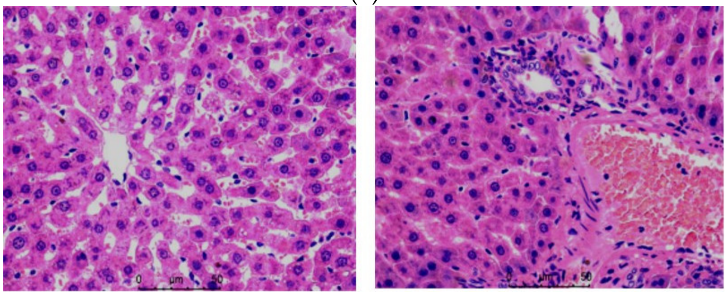

(**b**)

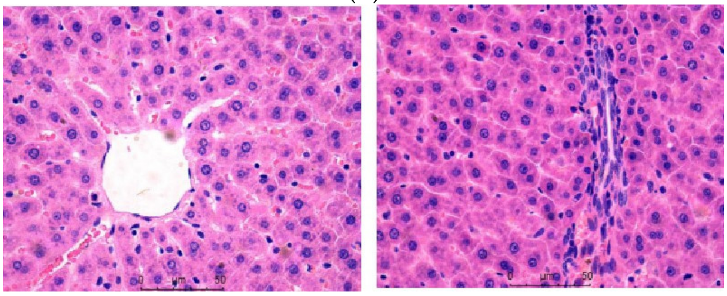

(**c**)

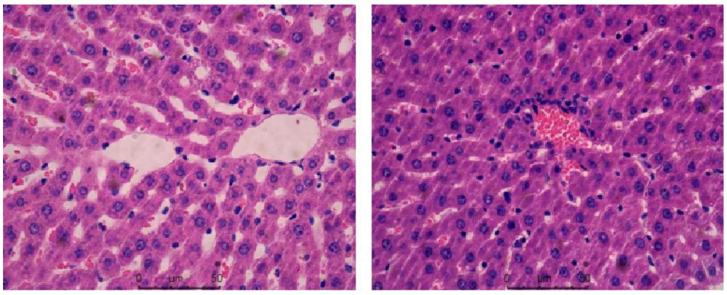

(**d**)

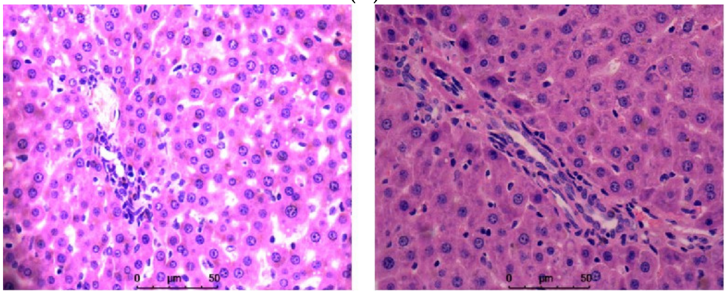

(**e**)

**Figure S4.** Histopathological analysis of liver sections after drug withdrawal (x 400): control group (**a**), PM-H (**b**), PM-M (**c**),PMP-H (**d**), PMP-M (**e**)
